# Iron status of schoolchildren (6–15 years) and associated factors in rural Nigeria

**DOI:** 10.3402/fnr.v59.26223

**Published:** 2015-05-06

**Authors:** Rufina N. B. Ayogu, Adaobi M. Okafor, Henrietta N. Ene-Obong

**Affiliations:** 1 Department of Home Science, Nutrition, and Dietetics, University of Nigeria, Nsukka, Nigeria; 2 Department of Biochemistry (Nutrition and Dietetics Unit), University of Calabar, Calabar, Nigeria

**Keywords:** anaemia, iron intake, parasitic infections, schoolchildren, rural area

## Abstract

**Background:**

Schoolchildren are vulnerable to anaemia because of their higher iron need to meet the demands of puberty and adolescence.

**Objective:**

The survey determined the haemoglobin levels of schoolchildren aged 6–15 years and the factors affecting their haemoglobin status.

**Design:**

Data were obtained through a cross sectional survey of 450 randomly selected schoolchildren in Ede-Oballa, Nsukka, Enugu State, Nigeria. Ninety were selected for clinical examination, biochemical tests, and nutrient intake study. Haemoglobin, malaria, and stool analysis were carried out by the cyanmethaemoglobin, thin blood film, and wet mount direct methods, respectively. Iron intake was determined by a three-day weighed food intake.

**Results:**

Results showed that the schoolchildren had pallor (35.6%), brittle hair (31.1%), koilonychia (2.2%), oedema (4.4%) and sore/smooth tongue (7.8%). The children also had malaria (58.9%) and *Entamoeba histolytica* (42.2%), hookworm (36.7%), tapeworm (35.6%), whipworm (34.5%), and roundworm (27.9%) infestations. Iron intake was inadequate (<100% of recommended nutrient intake) for most of the children. The mean haemoglobin levels of the schoolchildren were low. The 6–9, 10–12, and 13–15 year olds had 9.0, 9.1, and 9.3 g/dl, respectively. Most (85.5%) of them had anaemia. Moderate anaemia was prevalent in 62.2%. Severe anaemia affected the 6–9 year olds more. Malaria (*P<*0.001), *Entamoeba histolytica* (*P<*0.01), hookworm (*P<*0.05), tapeworm (*P<*0.01), and whipworm (*P<*0.001) caused significant reduction in haemoglobin level. Age (*b*=1.284, *P<*0.05), birth order (*b*=−0.629, *P<*0.01), frequency of illness attack (*b*=−1.372, *P<*0.01), household size (*b=*−0.526, *P<*0.05), and frequency of skipping breakfast (*b=*−1.542, *P<*0.001) were factors that influenced the haemoglobin status of the children.

**Conclusion:**

The schoolchildren had poor iron status as a result of consumption of plant sources of iron with low bioavailability, parasitic infections, birth order, skipping of breakfast, large household size, and frequent bouts of illnesses.

The causes of nutritional and health problems among schoolchildren are multidimensional. Inadequate intake and parasitic infections (particularly helminthiasis) are among the major causes. Intestinal helminths can use up to 25% of the food ingested by children (1). Hookworm and whipworm have been associated with intestinal bleeding. In most cases, nutrient intake may be adequate but the presence of intestinal helminths prevents maximum absorption of these nutrients by the body. In addition, most nutrient intakes are based on plant foods with low bioavailability. Ene-Obong et al. observed that all the meals consumed in school by adolescents in Enugu State, Nigeria, were plant based (2), which limits the biological value of the meals. A predominantly plant-based diet is one of the etiological factors behind some nutritional and health problems observed among school-aged children. It was not surprising therefore that 40 and 32% of adolescent males and females, respectively, were vitamin A deficient, whereas 47% of all adolescents were vitamin C deficient (2).

There is a wide range of practices that affect the health and nutritional status of schoolchildren. These practices include irregular hand washing, skipping meals, irregular use of footwear, not sleeping under insecticide-treated mosquito nets, and use of bushes for defecation. Some of these practices increase the preponderance of parasitic infections and a higher prevalence of nutrient deficiencies. The key nutrient deficiency observed among school-aged children is iron deficiency anaemia (3, 4). The Standing Committee on Nutrition (SCN) reported that the prevalence of iron deficiency anaemia among schoolchildren in Africa stood at 49.8% (3). Anaemia is an important public health problem. It has been associated with various nutrient deficiencies, bleeding (especially menstrual flow), and parasitic infections (5, 6). These nutrient deficiencies include low iron; zinc; copper; folic acid; vitamins C, E, A, and B_12_; riboflavin; thiamine; and pyridoxine.

Iron deficiency and anaemia are associated with poor growth and cognitive development, lowered immunity with increased risk to infectious diseases, and reduced work productivity. An unhealthy schoolchild is unlikely to have regular school attendance, good academic performance, and involvement in extracurricular activities.

Information on the iron status of school-aged children has not been well documented in Nigeria (4). This study was aimed at assessing the iron intake and haemoglobin status of school-aged children. It also determined factors that influenced the iron status. This study was undertaken to provide data necessary for the planning and implementation of positive strategies for the resolution of nutrition and health problems among schoolchildren, particularly those in the rural areas, where most (about 70%) Nigerians live (7) and which are devoid of infrastructure and quality educational opportunities.

## Materials and methods

### Study area

The study took place in Ede-Oballa, in the local government area of Nsukka, Enugu State, Nigeria.

### Calculation of sample size and sampling technique

The study population was made up of 2,366 free living schoolchildren aged 6–15 years. These children were drawn from all seven primary and three secondary schools in Ede-Oballa. The study employed a descriptive cross-sectional design. The sample size for this study was calculated using the formula

$$N=\frac{4P\left(1-P\right)}{W^{2}}$$

where *N*=total number of children required for the study, *P*=proportion of the respondents assumed to have abnormal iron status, and *W*=required precision level or probability level taken for this study (0.05 or 5%). The sample size by school, age, and sex was obtained by simple proportion (8).

SCN reported that the prevalence of iron deficiency anaemia among schoolchildren in Africa stood at 49.8% (3). Because there was no available national data on the prevalence of iron deficiency anaemia among schoolchildren in Nigeria, the prevalence rate in Africa was then used as the *P* value. This yielded a total of 450 schoolchildren required for this study. Twenty percent (90) of the sample size was selected as subsample for nutrient intake study, clinical examination, and biochemical tests. The sample was selected using multistage random sampling technique.

### Ethical approval and consent

Ethical approval was obtained from the Public Health/Primary Health Care Department of the Enugu State Ministry of Health, Enugu (MH/MSD/38). Informed consent was obtained from the parents or guardians of the participants. Oral consent was also obtained from the children. Children whose parents or guardians declined were replaced by balloting.

### Exclusion criteria

Exclusion criteria were inability to ascertain a child's age and refusal to participate.

### Data collection methods

#### Questionnaire

A validated and pretested questionnaire was used to obtain data from the pupils by interviewing the parent–child pairs.

#### Clinical examination

The schoolchildren were examined for signs of anaemia using an examination guide validated by the Department of Home Science, Nutrition, and Dietetics, University of Nigeria, Nsukka.

### Biochemical analyses

#### Haemoglobin determination

Venous blood (5 ml) was collected from each of the schoolchildren by a laboratory scientist. The cyanmethaemoglobin method was used to determine the blood haemoglobin level. The result was classified according to WHO/UNICEF/UNU (9).

#### Malaria parasite detection

The thin blood film method was used to detect the presence of malaria parasites. A positive result was recorded when different stages of growth of plasmodium were seen inside the erythrocytes.

#### Stool analysis

Stool samples were collected into coded sample bottles and analysed fresh using the wet mount direct method. The presence of ova/cysts was reported as positive.

### Statistical analysis

Data were analysed using the Statistical Product and Service Solution (SPSS, version 16.0). These were presented in frequencies and percentages. *T*-test and chi-square test were used to establish the relationship among variables. Data from the malaria test was analysed by ANOVA, and Duncan's new multiple range test was used to separate the group means. Multiple regression analysis was used in determining the factors influencing haemoglobin levels. Significance was accepted at the 95% confidence interval.

## Results

A total of 450 schoolchildren were involved in the study. Females constituted 51.6% and males 48.4% of the group. The ages of the children were as follows: 32.7% were 6–9 years old, 26.2% were 10–12 years old, and 41.1% were 13–15 years old. Primary school pupils accounted for 56.0% of the entire study group and those in secondary school accounted for 44.0%.

Table 1 shows the general characteristics of the schoolchildren. Forty-four percent of the schoolchildren had a household size of 5–8 people; 33.6% had 1–4, and 22.4% had 9 or more people in their households. The most common birth order was 5–8 (40.7%); 35.1% were 1–4, whereas 24.2% were 9th or greater. A total of 38.0% children used a pit latrine; 34.3% used the bush and only 23.5% used a water closet. Clinical symptoms of illness often experienced by the children were tiring easily (65.8%), feeling cold easily (55.1%), decreased ability to concentrate (34.9%), often feeling dizzy (39.3%), and headaches (21.3%). More than half (51.6%) of the schoolchildren had attacks of illness up to two times in 6 months. A few (10.0%) became sick three times in 6 months. About 45.0% received treatment from chemist shops (patent medicine dealers). Only 37.3% resorted to hospitals/health centres for the treatment of illnesses. More than half (56.2%) of the children skipped breakfast. Breakfast was skipped by 1.8% of the children daily, by 7.3% of the children 1–3 times a week, by 5.1% 4–6 times weekly, and by 42.0% occasionally.

**Table 1:** General characteristics of the schoolchildren

Variables	6–9 years *N* (%)	10–12 years *N* (%)	13–15 years *N* (%)	Total *N* (%)
Household size (persons) 1–4 5–8 9 and above Total	66 (44.9) 47 (32.0) 34 (23.1) 147 (100.0)	39 (33.1) 55 (46.6) 24 (20.3) 118 (100.0)	46 (24.9) 96 (51.9) 43 (23.2) 185 (100.0)	151 (33.6) 198 (44.0) 101 (22.4) 450 (100.0)
Birth order First–fourth Fifth–eighth Ninth and above Total	52 (35.3) 57 (38.8) 38 (25.9) 147 (100.0)	47 (38.9) 45 (38.1) 26 (22.0) 118 (100.0)	59 (31.9) 81 (43.8) 45 (24.3) 185 (100.0)	158 (35.1) 183 (40.7) 109 (24.2) 450 (100.0)
Type of toilet in use Pit Bucket Water closet Bush Total	55 (37.4) 6 (4.0) 53 (36.1) 33 (22.5) 147 (100.0)	45 (38.1) 4 (3.4) 39 (33.1) 30 (25.4) 118 (100.0)	71 (38.3) 9 (4.9) 14 (7.6) 91 (49.2) 185 (100.0)	171 (38.0) 19 (4.2) 106 (23.5) 154 (34.3) 450 (100.0)
Symptoms of illness often experienced (MR) Gets cold easily Decreased ability to concentrate Gets tired easily Has frequent bouts of headache Often feels dizzy Has difficulty seeing at night	84 (18.6) 55 (12.2) 106 (23.5) 30 (6.6) 58 (12.9) 1 (0.2)	82 (18.2) 49 (10.9) 97 (21.6) 32 (7.1) 59 (13.1) 1 (0.2)	82 (18.3) 53 (11.8) 93 (20.6) 34 (7.6) 60 (13.3) 1 (0.2)	248 (55.1) 157 (34.9) 296 (65.8) 96 (21.3) 177 (39.3) 3 (0.6)
Frequency of illness attack within a 6-month period Once Twice Three times More than three times Total	57 (38.8) 73 (49.7) 11 (7.5) 6 (4.0) 147 (100.0)	41 (34.8) 58 (49.1) 16 (13.6) 3 (2.5) 118 (100.0)	65 (35.1) 101 (54.6) 18 (9.7) 1 (0.6) 185 (100.0)	163 (36.2) 232 (51.6) 45 (10.0) 10 (2.2) 450 (100.0)
Place of treatment of illnesses Hospital/health centre Chemist shop Herbalist Prayer house None Total	55 (37.4) 60 (40.8) 19 (12.9) 11 (7.5) 2 (1.4) 147 (100.0)	42 (35.6) 61 (51.7) 7 (5.9) 8 (6.8) 0 (0.0) 118 (100.0)	71 (38.3) 83 (44.9) 20 (10.8) 11 (6.0) 0 (0.0) 185 (100.0)	168 (37.3) 204 (45.3) 46 (10.2) 30 (6.7) 2 (0.5) 450 (100.0)
Frequency of skipping breakfast weekly Daily One to three times Four to six times Occasionally Never skipped breakfast Total	1 (0.7) 20 (13.6) 10 (6.8) 47 (32.0) 69 (46.9) 147 (100.0)	2 (1.7) 4 (3.4) 5 (4.2) 38 (32.2) 69 (58.5) 118 (100.0)	5 (2.7) 9 (4.9) 8 (4.3) 104 (56.2) 59 (31.9) 185 (100.0)	8 (1.8) 33 (7.3) 23 (5.1) 189 (42.0) 197 (43.8) 450 (100.0)

MR=Multiple response.

**Fig. 1:**
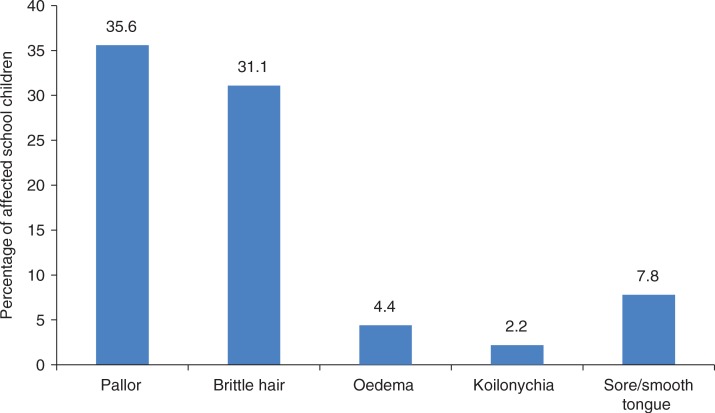
Signs of anaemia exhibited by the school children.

Figure 1 shows the signs of anaemia observed in the children. Pallor was found in 35.6% of the schoolchildren; 31.1% had brittle hair. Several (2.2, 4.4, and 7.8%) had koilonychia, oedema, and sore/smooth tongue, respectively.

**Fig. 2:**
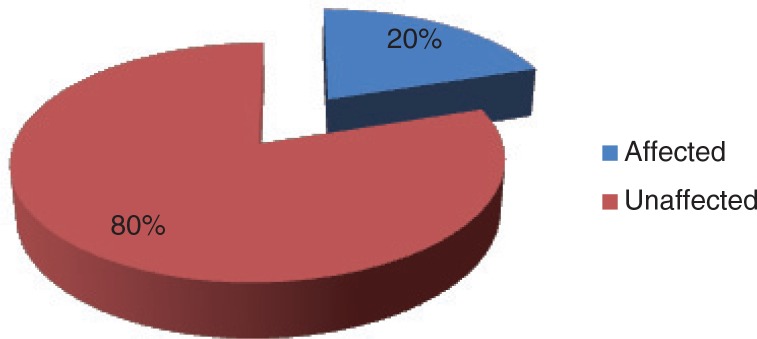
Prevalence of parasitic infections among the schoolchildren.

Figure 2 presents the prevalence of parasitic infections among the schoolchildren. Most (80.0%) of the schoolchildren were affected by at least one form of parasitic infection. Only 20.0% were free from any form of parasitic infection.

**Fig. 3:**
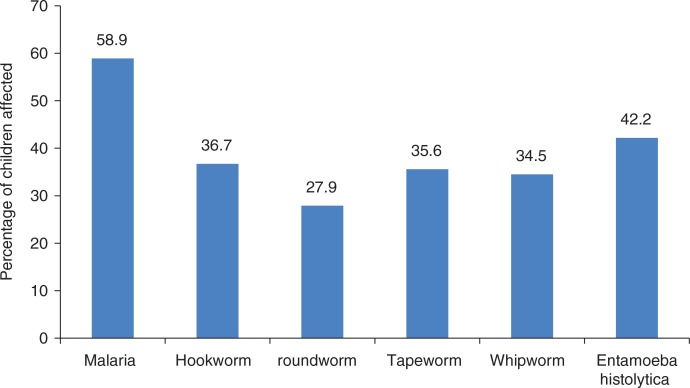
Percentage of the children affected by different species of parasitic infections.

Figure 3 shows the percentage of children affected by different species of parasitic infections. Malaria affected 58.9% of the schoolchildren; 42.2% were affected by *Entamoeba histolytica*. Hookworm and tapeworm infestations affected 36.7 and 35.6% of the children, respectively; and 34.5 and 27.9% had whipworm and roundworm, respectively.

Table 2 presents the mean haemoglobin, iron intake, and percentage contribution of iron intake to recommended nutrient intake (RNI) of the schoolchildren by age and sex. Mean haemoglobin levels of the 6–9, 10–12, and 13–15 year olds were 9.0, 9.1, and 9.3 g/dl, respectively. Males of 6–9 and 10–12 years had slightly better mean haemoglobin than their female counterparts. The lowest value (8.4 g/dl) was observed among females aged 6–9 years. Iron intake was adequate for 6–9-year-old males (103.1% of RNI) and for both males and females aged 10–12 years (117.5 and 105.0% of RNI, respectively). The 13–15-year-old male and female children had the lowest intake, providing only 65.8 and 64.8% of their iron RNI, respectively.

**Table 2:** Mean haemoglobin, iron intake, and percentage contribution to recommended nutrient intake (RNI) of the schoolchildren

Age (years)/sex		*N* (%)	Haemoglobin (g/dl)	Iron intake (mg)	Percentage contribution of intake to iron RNI
6–9	Male	14 (43.7)	9.5±2.03	16.5±6.931	103.1
	Female	18 (56.3)	8.4±2.00	15.2±4.619	95.0
Group mean		32 (100.0)	9.0±2.04	15.8±5.683	
10–12	Male	13 (52.0)	9.3±1.31	18.8±5.105	117.5
	Female	12 (48.0)	8.8±2.08	16.8±5.580	105.0
Group mean		25 (100.0)	9.1±1.71	17.8±5.324	
13–15	Male	14 (42.4)	9.2±1.22	15.8±6.594	65.8
	Female	19 (57.6)	9.3±1.45	17.5±7.498	64.8
Group mean		33 (100.0)	9.3±1.34	16.8±7.073	

Table 3 illustrates the haemoglobin status of the schoolchildren by age. Moderate anaemia was prevalent in 62.2% of the children. Children aged 13–15 years (66.7%) were affected by moderate anaemia much more than those in other age groups. Severe anaemia (15.6%) affected the 6–9 year olds more than others. Haemoglobin was significantly (*P<*0.001) associated with age.

**Table 3:** Prevalence of anaemia among the schoolchildren by age

Variables	6–9 years *N* (%)	10–12 years *N* (%)	13–15 years *N* (%)	Total *N* (%)
Haemoglobin (g/dl)				
Normal (≥11.5/12.0)	9 (28.1)	4 (16.0)	0 (0.0)	13 (14.5)
Mild anaemia (10.0–11.4/11.9)	0 (0.0)	2 (8.0)	11 (33.3)	13 (14.5)
Moderate anaemia (7.0–9.9)	18 (56.3)	16 (64.0)	22 (66.7)	56 (62.2)
Severe anaemia (<7.0)	5 (15.6)	3 (12.0)	0 (0.0)	8 (8.8)
Total	32 (100.0)	25 (100.0)	33 (100.0)	90 (100.0)

χ^2^=27.587, df = 6, *P*=0.000 (*P*<0.001).

Table 4 shows the effect of parasitic infections on the haemoglobin level of the children. Haemoglobin was significantly reduced in schoolchildren with malaria (*P<*0.001), hookworm (*P<*0.05), tapeworm (*P<*0.01), whipworm (*P<*0.001), and *Entamoeba histolytica* (*P<*0.01). Children without these parasites had higher haemoglobin levels.

**Table 4:** Effect of parasitic infections on haemoglobin

Parasitic infections	Haemoglobin (g/dl)	Level of significance
Malaria parasite count		
Zero count (negative)	9.9^a^	*F*=9.364, *P*=0.000***
One +	9.3^b^	
Two ++	8.5^bc^	
Three +++	7.6^c^	
Hookworm		
Negative	9.3±1.67	*T*=−2.221, *P*=0.029*
Positive	8.5±1.67	
Roundworm		
Negative	9.0±1.68	*T*=0.693, *P*=0.490
Positive	9.3±1.78	
Tapeworm		
Negative	9.5±1.54	*T*=−3.278, *P*=0.001**
Positive	8.3±1.76	
Whipworm		
Negative	9.6±1.61	*T*=−4.340, *P*=0.000***
Positive	8.1±1.46	
*Entamoeba histolytica*		
Negative	9.5±1.71	*T*=−3.017, *P*=0.003**
Positive	8.4±1.54	

Malaria: values with different superscripts are significantly different.

* *P<*0.05

** *P<*0.01

*** *P<*0.001.

Table 5 shows the relationship between the clinical symptoms of illness often experienced by the children and their haemoglobin levels. Haemoglobin decreased in the presence of these symptoms. The relationships with cold (*P<*0.01) and dizziness (*P<*0.05) were significant.

**Table 5:** Relationship between the clinical symptoms often experienced and haemoglobin

Clinical symptoms	*N* (%)	Haemoglobin (g/dl)	Level of significance (*T*-test)
Cold			
Yes	48 (53.3)	8.5±1.54	−3.394, 0.001**
No	42 (46.7)	9.7±1.69	
Decreased ability to concentrate			
Yes	55 (61.1)	8.8±1.73	−1.503, 0.136
No	35 (38.9)	9.4±1.63	
Tiredness			
Yes	56 (62.2)	8.8±1.76	−1.985, 0.050
No	34 (37.8)	9.5±1.53	
Headache			
Yes	36 (40.0)	8.8±1.69	−1.277, 0.205
No	54 (60.0)	9.2±1.71	
Dizziness			
Yes	57 (63.3)	8.8±1.67	−2.251, 0.027*
No	33 (36.7)	9.6±1.66	

* *P*<0.05

** *P*<0.01.

The regression coefficients of variables affecting the haemoglobin levels of the schoolchildren are shown in Table 6. Age (*b*=1.284, *P<*0.05), birth order (*b*=−0.629, *P<*0.01), frequency of illness attack (*b*=−1.362, *P<*0.01), frequency of skipping breakfast (*b*=−1.542, *P<*0.001) and household size (*b*=−0.526, *P<*0.05) were the variables with a significant relationship with haemoglobin.

**Table 6:** Factors affecting haemoglobin status of schoolchildren

Variables	Multiple regression coefficients	Level of significance
Age	1.284*	0.013
Birth order	−0.629**	0.004
School level	−0.344	0.312
Frequency of illness attacks	−1.372**	0.005
Household size	−0.526*	0.036
Frequency of skipping breakfast	−1.542***	0.000
Iron intake	0.425	0.117

* *P*<05

** *P*<0.01

*** *P*<0.001.

## Discussion

The percentage of children who skipped breakfast (Table 1) is worrisome and is in line with the report of Ene-Obong et al. (2). This missing meal may be responsible for the inadequate iron intake observed in some of the schoolchildren. It was also observed during the 3-day nutrient intake study that iron intake was derived mainly from plant sources. Most animal sources were out of reach for most of the schoolchildren due to cost. Plant sources of iron have lower bioavailability than animal sources because antinutrients in them limit iron bioavailability. However, although the positive relationship of iron intake with haemoglobin level was not significant (*b*=0.425, *P*>0.05), it nonetheless did imply that the higher the iron intake, the better the iron status.

Schoolchildren who go to school on an empty stomach are unlikely to learn anything. Hunger is a major cause of undernutrition. Every year, hunger and undernutrition claim more than 10 million lives – more than the deaths from AIDS, malaria, and tuberculosis combined (5). The effects of poor nutrition are difficult to identify because most of these effects are due to hidden hunger – a condition in which vital micronutrients such as iron are deficient.

The large household sizes recorded in this study (Table 1) imply lack of adherence to family planning due to cultural factors. Birth order has been shown by some researchers (10, 11) to affect the nutritional status of individuals. It was therefore not surprising that haemoglobin levels decreased as birth order increased (*P<*0.01). Horton observed that the long-run outcome is far from equitable because children born later are born when per capita resources in families are smaller (12). The effects of birth order on nutritional status could be a result of strain on household resources, because having more children increases household size. This effect is worsened in cases where household income is also low.

Our findings regarding the types of toilet in use (Table 1) agreed with the report of Maziya-Dixon et al. (7) that the pit toilet was the most commonly used in Nigeria, followed by the bush method. This practice implies an increased tendency toward food and water contamination, with an increase in the prevalence of worm infestations and higher chances of reinfection even after treatment.

The heavy patronage of chemist shops (Table 1) for treatment of malaria and other infections and illnesses, rather than hospitals and health centres, is a cause for concern. We attribute this propensity for patronizing chemist shops to ignorance and poverty; it implies inadequate treatment of health conditions, with the danger of recurrence, long-term complications, and in the long run waste of resources. It was therefore not surprising that a little more than half of the children had attacks of illness up to two times in 6 months. Inadequate treatment of ailments as a result of heavy patronage of chemist shops could also have been responsible for the frequent bouts of illness reported here. Another implication is that families’ limited financial resources will continue to be diverted to treatment of ailments rather than other gainful activities such as good food, housing, and education.

Almost all (80%) of the schoolchildren had at least one form of parasitic infection (Fig. 2). This finding is in line with the work done by Onimawo et al. (4). These authors reported that 84.9% of all the children examined had ova and cyst of intestinal parasites in their stools. The high percentage of the study population that had parasitic infections was attributed to reinfection or inadequate treatment, unavailability of insecticide-treated mosquito nets, and poor personal and environmental sanitation. Most of these children went about without footwear in a community where 34.3% of the households defecated in the bushes. It has been shown that most intestinal helminths are soil helminths that gain entrance into the human body through food or water contamination or by piercing the skin (13, 14). Blood loss due to these infestations can be alarming, especially when prolonged. Such blood loss might have contributed to the high prevalence of anaemia reported in this study. Malaria, hookworm, tapeworm, whipworm, and *Entamoeba histolytica* caused a significant (*P<*0.05) decrease in the haemoglobin levels of the children (Table 4). The lack of significant relationship between roundworm and haemoglobin was probably due to a light load of this parasite in the intestine. Heavy parasitic (of all parasites) load has been reported (15) to cause malabsorption of iron as well as of other food nutrients, because the worms live in the duodenum and jejunum where nutrient absorption occurs.

The symptoms of illness often experienced by these children (Table 1) were similar to those observed by Hong and Hwang (16) and Hong, Cho, and Chung (17). Tiredness, dizziness, decreased ability to concentrate, and in some cases headache indicate anaemia (18, 19) because circulating haemoglobin is unable to carry oxygen to the cells for adequate tissue oxygenation. Increased susceptibility to cold is a feature of iron deficiency because iron deficiency interferes with the body's ability to regulate temperature (20).

The low mean haemoglobin of the schoolchildren (Table 2) is a cause of great concern. The values fell within the category of moderate anaemia (7.0–9.9 g/dl). This finding was attributed mainly to inadequate iron intake and parasitic infections. The implication of this finding is enormous. Haemoglobin is the pivot of energy metabolism because of its oxygen-carrying and distribution capacity. The low values observed imply reduced work capacity, lowered immunity, and other grave consequences of anaemia.

The high prevalence of anaemia (85.5%) observed in this study (Table 3) was not a surprise. Onimawo et al. (4) reported an equally high prevalence of anaemia (82.6%) among schoolchildren of 7–12 years in rural communities of Abia State, Nigeria. The higher prevalence (100.0%) of anaemia among the 13–15-year-olds was attributed to age. Adolescence is a period when iron requirement is increased to compensate for losses due to menstruation and demand for rapid growth. This fact explains the finding that moderate anaemia increased significantly (*P<*0.001) with age. The observed severe anaemia among the 6–9 year olds might be due to higher prevalence of malaria, trichuriasis, and *Entamoeba histolytica* among them. Pallor, brittle hair, oedema, sore/smooth tongue, and koilonychia have been associated with anaemia (18, 19). It was therefore not surprising to observe that these signs had negative associations with haemoglobin.

The relationship that existed between clinical symptoms of illness often experienced by the children and their haemoglobin levels (Table 5) agreed with the report of Hong et al. when iron status is low, the clinical symptoms of tiredness, feeling cold, dizziness, headache, and decreased ability to concentrate occur at a higher rate (17). All the symptoms of illness often experienced by the children had negative associations with haemoglobin. However, the lack of significant association of haemoglobin with decreased ability to concentrate, tiredness, and headache may be due to the level of haemoglobin of the affected children. The manifestations of clinical symptoms depend on the severity of the condition. The lower the haemoglobin level is, the more symptoms are exhibited. In this study, the mean haemoglobin levels of the children were all within the range of moderate anaemia.

Birth order, frequency of illness attacks, household size, and frequency of skipping breakfast were factors that contributed to the prevalence of anaemia reported in this study (Table 6). There was a very slight increase in haemoglobin as age increased. But as birth order, frequency of bouts of illness, frequency of skipping breakfast, and household size increased, haemoglobin decreased significantly (*P<*0.05).

## Conclusion

Anaemia is a severe public health problem among rural schoolchildren in Nigeria. This ugly situation was a function of parasitic infections, high birth order, large household size, frequent bouts of illnesses, skipping of breakfast, and low consumption of animal sources of iron.

## Recommendation

Restructuring of school health programme with emphasis on school meals, health and nutrition education, regular deworming exercises, and regular health supervision of the schoolchildren are recommended as measures to curb this condition and enable the children to benefit maximally from their education.

## References

[1] UNICEF (2011). An overview of nutritional status of Nigerians.

[2] Ene-Obong HN, Odoh IF, Ikwuagwu OE (2003). Plasma vitamin A and C status of in-school adolescents and associated factors in Enugu State, Nigeria. J Health Popul Nutr.

[3] Standing Committee on Nutrition (SCN) (2002). School-age children: their health and nutrition, No 25.

[4] Onimawo LA, Ukegbu PO, Asumugha VU, Anyika JU, Okudu H, Echendu CA (2010). Assessment of anaemia and iron status of school age children (aged 7–12 years) in rural communities of Abia State, Nigeria. Afr J Food Agric Nutr Dev.

[5] Kraemer K, Zimmermann MB (2007). Nutritional anaemia.

[6] Oguntona T, Omojekun SO, Aminu FT, Demehin KO, Falana AO (2005). Federal Ministry of Health national guidelines on micronutrient deficiencies control in Nigeria.

[7] Maziya-Dixon B, Akinyele IO, Oguntona EB, Nokoe S, Sanusi RA, Harris E (2004). Nigeria food consumption and nutrition survey 2001–2003: summary.

[8] Lind DA, Mason RD, Marchal WG (2000). Basic statistics for business and economics.

[9] WHO/UNICEF/UNU (2001). Iron deficiency anaemia: assessment, prevention and control: a guide for programme managers. (WHO/NHD/01.3).

[10] Sommerfelt AE, Kathryn S (1994). Children's nutritional status: DHS comparative studies.

[11] Jeyaseelan L, Lakshman M (1997). Risk factors for malnutrition in South Indian children. J Biosoc Sci.

[12] Horton S (1988). Birth order and child nutritional status: evidence from the Philippines. Chic J.

[13] Lucas AO, Gilles HM (2003). Short textbook of public health medicine for the tropics.

[14] Allen C, Ajello CA (2009). The Honduras children's micronutrient and deworming project. Sight Life Mag.

[15] Thurnham D, Northrop-Clewes C, K Kraemer, MB Zimmermann (2007). Infection and the aetiology of anaemia. Nutritional anaemia.

[16] Hong SM, Hwang HJ (2003). Effects of nutrition education and iron supplementation on iron nutrition and anaemia of middle school girls. J Food Sci Nutr.

[17] Hong S, Cho J, Chung H (2007). Iron status, clinical symptoms and anthropometry between normal and anaemic groups of middle age school girls. Nutr Res Pract Spring.

[18] Conrad ME (2006). Iron deficiency anaemia.

[19] Marieb EN, Hoehn K (2007). Human anatomy and physiology.

[20] Yip R, BA Bowman, RM Russell (2001). Iron. Present knowledge in nutrition.

